# Systemic lupus erythematosus with podocyte infolding glomerulopathy: A case report and literature review

**DOI:** 10.1097/MD.0000000000039809

**Published:** 2024-10-25

**Authors:** Huiqing Zhang, Jie Lin, Hanqi Lu, Yunliang Zhong, Lie Deng, Bin Kuang, Qiang Li

**Affiliations:** a Guangzhou University of Chinese Medicine, Dongguan Hospital of Traditional Chinese Medicine, Dongguan, Guangdong 523000, China.

**Keywords:** case report, diagnostic criteria, pathogenesis, pathological characteristics, podocyte infolding glomerulopathy

## Abstract

**Rationale:**

Podocyte infolding glomerulopathy (PIG) is a rare glomerular disease, its diagnosis mainly depends on pathological manifestations of the kidney. Few clinical cases of PIG have been reported, but it is sometimes associated with connective tissue diseases. Here we describe a case of systemic lupus erythematosus (SLE) with PIG and undertake a review of the literature.

**Patient concerns:**

A 34-year-old female patient was admitted to our hospital in August 2019 with repeated facial erythema and proteinuria for more than 10 years. The patient was previously diagnosed with SLE.

**Diagnosis:**

Systemic lupus erythematosus.

**Interventions:**

Renal biopsy was performed to investigate ongoing proteinuria and the results were consistent with PIG. Treatment with methylprednisolone, hydroxychloroquine sulfate, mycophenolate mofetil, and candesartan ester.

**Outcomes:**

Improved the patient’s condition and resolved the proteinuria.

**Lessons:**

This study reported a case of PIG and SLE. The patient was diagnosed according to biopsy, and the disease remain stable after immunosuppressive therapy. It is recommended to carefully study renal biopsies from patients with proteinuria and underlying autoimmune diseases to identify additional cases.

## 1. Introduction

Podocyte infolding glomerulopathy (PIG), a pathological condition characterized by unique ultrastructural findings, was discovered in 1992 when Sato et al^[1]^ reported on 5 renal biopsy specimens using electron microscopy (EM). These findings include the inversion and folding of podocytes into the glomerular basement membrane (GBM), which appear as microspheres and microtubules under EM. Initially, PIG was considered to be a variant of membranous nephropathy (MN), which is characterized by annular subepithelial deposition. However, in 2008, PIG was recognized as a possible distinct disease entity.^[2]^

Relatively few cases of PIG have been reported in the literature, some of which are associated with connective tissue diseases, including systemic lupus erythematosus (SLE). However, it is not clear whether PIG is a distinct disease in these patients or if it relates to co-existing diagnoses.^[3]^ Therefore, this study aims to report a case of a 34-year-old woman with SLE and PIG, and review relevant literature reporting clinical cases of PIG.

## 2. Case presentation

A 34-year-old Chinese woman was admitted to our hospital in August 2019 with recurrent facial erythema and proteinuria persisting for more than 10 years. On physical examination, the patient exhibited flaky light erythema on both cheeks without flaking or itching. Laboratory examination revealed no abnormalities in blood routine and virus infection. Immunoassay testing yielded the following results: antinuclear antibody (ANA) > 500 AU/mL (normal range: 0–32), anti-double-stranded DNA antibody 36.1 IU/ml (normal range: 0–24), anti-SSA/Ro52 antibody positive (+), anti-SSA/Ro60 antibody positive (+), anti-U1-snRNP antibody positive (+). Urinalysis showed protein (2+) and microalbumin > 0.15 g/L. Urinary protein excretion was 446 mg/d and urine protein creatinine ratio was 0.05.

The patient had previously been diagnosed with SLE for 10 years and had been undergoing long-term medication, including prednisone, hydroxychloroquine sulfate, methotrexate, and azathioprine. Nine months ago, she was hospitalized in the rheumatology department of our hospital, and the treatment regimen was changed to methylprednisolone 20 mg once daily, hydroxychloroquine sulfate 0.2 g twice daily, and merti-mescaline dispersible tablets 0.75 g. Since then, the patient’s condition has stabilized and she was discharged from the hospital. Regular outpatient reviews showed persistent urinary protein levels ranging from 1+ to 3+ levels. The patient denied any family history.

**Figure 1.**
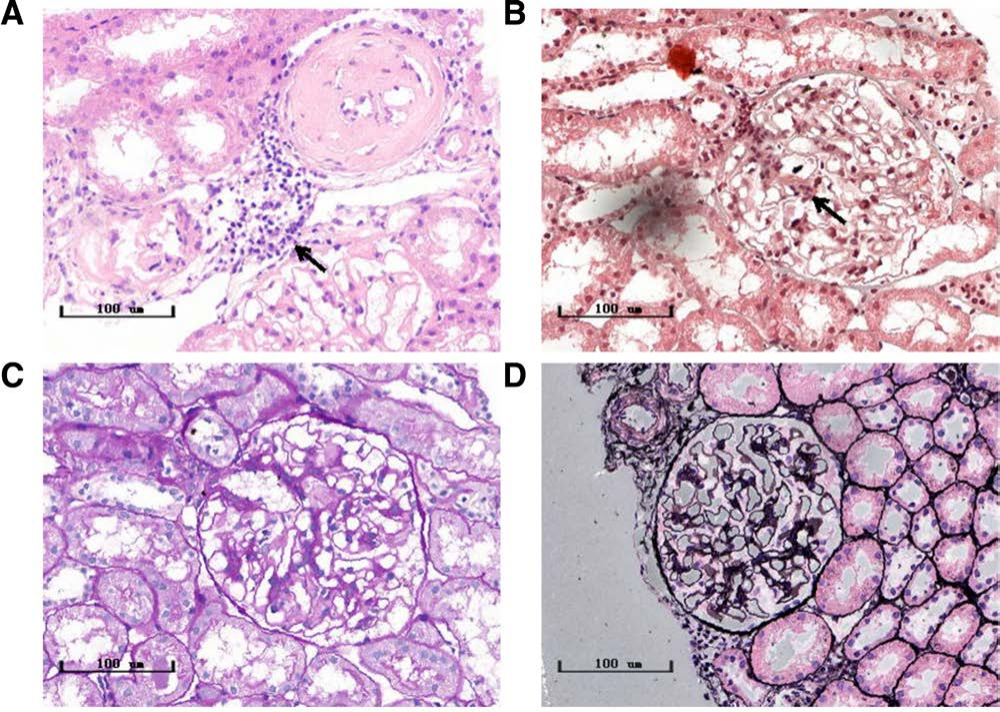
Histological analysis of the kidney biopsy. The light microscope specimens were stained with (A) Hematoxylin and Eosin (HE); (B) Masson; (C) Periodic acid–Schiff (PAS); (D) Periodic Schiff–Methenamine Silver (PASM).

**Figure 2.**
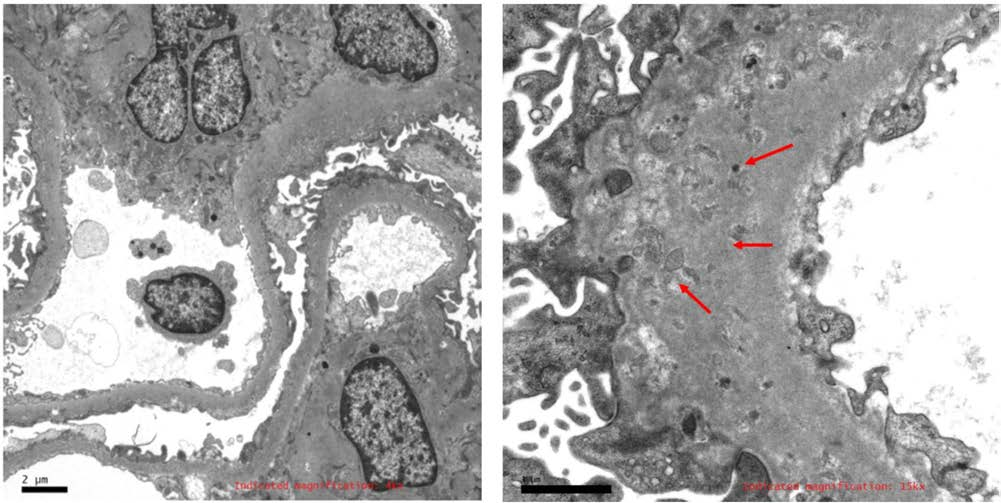
The electron microscope specimen was stained with toluidine blue, and 2 glomeruli were seen. More microspheres or microcysts are seen nearby, the capillary collaterals are open, the wall cells are not proliferating, the basement membrane is irregularly thickened, and the peduncles are mostly united.

As the previous treatment regimen for SLE was not effective it was suggested that the case undergo a kidney biopsy. The results of the kidney biopsy were as follows: The volume of the glomeruli increased slightly, with slight proliferation of mesangial cells. The capillary loops were open, but the basement membrane appeared thickened and relatively rigid, with no obvious spike-like structure. There were no fuchsinophilic protein deposits in the mesangial area and subendothelial area, but deposits were found under the epithelium. No mesangial insertion or double track formation was observed. Proliferation of parietal epithelial cells and crescents was observed. Renal tubular epithelial cells exhibited granular and vacuolar degeneration, with small focal atrophy (approximately 5% of the area). There was small focal inflammatory cell infiltration in the renal interstitium, with no obvious fibrosis. The arterial wall showed thickening of the intima and narrowing of the lumen (**Fig. 1**). No significant regularity in immunoglobulin staining was observed. The EM specimen was stained with toluidine blue, revealing 2 glomeruli. Observed under the ultrathin section electron microscope, the capillary loops were open, parietal cells did not proliferate, and the basement membrane was slightly irregularly thickened, exceeding 800 nm in thickness. Fusion of most foot processes was observed, along with the presence of microspheres or microcapsules near the epithelial side of the basement membrane. Mesangial cells and matrix showed slight proliferation, with no exact electron-dense deposits found under the epithelium, mesangial area, or endothelium. Renal tubular epithelial cells exhibited vacuolar degeneration, while the renal interstitium showed no special lesions (**Fig. 2**). These characteristics led to the diagnosis of PIG in this case.

**Figure 3.**
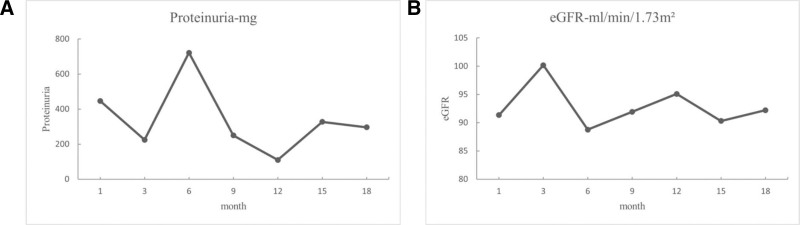
Follow-up monitoring of renal function and urinary protein. (A) Changes in eGFR over time (months); (B) changes in proteinuria with time (months).

The patient received methylprednisolone 12 mg once daily, hydroxychloroquine sulfate 0.2 g twice daily, mycophenolate mofetil dispersible tablets 0.75 g twice daily, and candesartan ester 2 mg once daily as treatment, which was maintained until March 2020. Starting March, 2020, the corticosteroid dosage was reduced to 8 mg/d, and mycophenolate mofetil dispersible tablets were changed to 0.5 g. The patient was followed up regularly for over 1 year. Her condition improved, with Creatinine at 49.8 (normal range: 41–73 μmol/L), cystatin at 0.67 (normal range: 0.54–1.15 mg/L), urea at 3.3 (normal range: 2.6–7.5 mmol/L), and no abnormalities in blood routine. Blood pressure and renal function were within normal ranges (**Fig. 3A**), and urinary protein was <500 mg/d (**Fig. 3B**).

Written consent for publication of this case report was obtained from the patient.

## 3. Discussion

This study reported a case of PIG in a female patient with SLE. The patient had a history of SLE with facial erythema and proteinuria for over ten years, but the treatment regimens with prednisone and hydroxychloroquine sulfate had been unsuccessful in resolving all symptoms. The patient underwent a kidney biopsy, revealing characteristics of PIG. She was treated with methylprednisolone, hydroxychloroquine sulfate, mycophenolate mofetil, and candesartan ester, which improved the patient’s condition and resolved the proteinuria.

The diagnosis of PIG in this case was based on the diagnostic criteria for PIG that were put forward by the Japanese Society of Nephrology (JSN), which established an expert group on PIG and analyzed data of 25 cases of PIG reported in Japan.^[2]^ These criteria include the presence of non-silvered vacuoles similar to those found in MN in the GBM under light microscopy and the presence of microsphere or microtubule structures of 50 to 150 nm within the GBM visible under EM.^[2]^ However, since the diagnosis of PIG relies solely on the morphologic manifestations of EM, it is still unclear whether PIG should be regarded as a distinct disease entity.

It has been observed that the majority of cases with PIG have underlying lesions, while some exhibit only mild lesions with few specific changes seen on light microscopy. The light microscopy appearance of PIG is similar to that of MN, and is characterized by a stiff, thickened GBM with non-silver-loving vacuoles. However, it can be differentiated from MN by fluorescence and EM. Masuda et al^[4]^ retrospectively analyzed 126 renal biopsies of primary MN and suggested that it is necessary to exclude cellular debris and virus-like particles to differentiate them from MN before diagnosing PIG, especially when microstructures are present in the GBM.

A literature review was conducted to comprehend the pathological characteristics and underlying causes of PIG. PubMed was searched using the keywords “podocyte infolding glomerulopathy.” Studies that did not report on treatment or prognosis were excluded. From the available literature, it was found that all 32 diagnosed cases of PIG so far originated from Asia, with a higher prevalence among (**Table 1**). The most frequently associated condition reported was SLE (16/32, 50.0%), while other diseases included tumors, chronic thyroiditis, hydronephrosis, hepatitis B virus infection, and dry syndrome. EM consistently revealed GBM thickening, fusion or loss of peduncles, and the formation of microspheres or microtubules in most cases (**Table 2**). Immunofluorescence staining was positive for G and C3 in the majority of patients, with only 9 cases being completely negative. These 9 cases included 5 cases of SLE, 2 cases of thyroid disease, 1 case of primary Sjögren’s syndrome (pSS), and 1 case of multiple myeloma. No significant regularity in immunoglobulin staining was observed. Our patient in this case also had a fully negative result, possibly due to prior immunotherapy.

**Table 1:** Clinical profiles of the PIG cases reported in the literature.

Case No	Sex	Age	Comorbidity	Cr (µmol/L)	Proteinuria	Therapy	Prognosis
1^2^	M	31	SLE	168	0.5 g/d	PSL	CR
2^2^	F	37	SLE	106.1	1 g/d	PSL, MMF	CR
3^2^	F	40	SLE	44.2	1.5 g/d	PSL	NR
4^2^	F	30	SLE	44.2	1.6 g/d	PSL	CR
5^2^	F	61	SLE, Takayasu arteritis	79.6	1.7 g/d	PSL, CsA	PR
6^2^	F	29	SLE, hydronephrosis	61.9	1.6 g/d	PSL	PR
7^2^	F	46	SLE, hydronephrosis	44.2	0.6 g/d	PSL	PR
8^2^	F	27	SLE	35.4	2.7 g/d	PSL, MMF	CR
9^2^	M	53	SLE, bilateral urethral stone	79.6	3.1 g/d	PSL	PR
10^2^	F	23	SLE	44.2	1.8 g/d	PSL	CR
11^2^	F	31	SLE	79.6	0.5 g/d	PSL	CR
12^2^	F	24	SLE, SS	53	6.0 g/d	PSL	CR
13^2^	M	49	PBC, SS, cystitis, SLE	97.2	2.2 g/d	PSL	CR
14^2^	F	20	SLE?	123.8	1.4 g/d	PSL	CR
15^2^	F	47	RA, pSS	53	1.3 g/d	PSL	PR
16^2^	F	51	pSS	53	3.7 g/d	PSL	CR
17^2^	F	30	MCTD	79.6	0.3 g/d	PSL	NR
18^2^	F	54	Basedow disease	221	6.0 g/d		PR
19^2^	F	57	Hypothyroidism, chronic thyroiditis	53	0.3 g/d		CR
20^2^	M	45	Absent	61.9	2.6 g/d	PSL	PR
21^2^	F	42	Ovarian mature teratoma	68.1	7.5 g/d	ARB	PR
22^2^	F	69	Absent	79.6	1.6 g/d		PR
23^2^	M	46	HBV infection	106.1	4.0 g/d	Diuretics	PR
24^2^	M	59	Tumor lysis syndrome	450.8	0.6 g/d	PSL	NR
25^2^	F	45	Absent	70.7	1.5 g/d	PSL	CR
26^13^	F	14	Absent	48.6	2.4 g/d	PSL	CR
27^14^	M	79	Multiple myeloma	113.2	1.4 g/d	PSL	Absent
28^15^	F	44	Absent	39.8	0.3 g/d	PSL, Kanarb	CR
29^16^	F	45	UCTD	145.9	5.8 g/d	PSL, MMF, RTX	PR
30^17^	F	27	pSS	168	0.6 g/d	PSL, HCQ	Lost
31^17^	F	23	SLE	47	16.8 g/d	PSL, HCQ, MMF	CR
32	F	34	SLE	48.3	0.4 g/d	MePr, HCQ, MMF	CR

ARB = angiotensin receptor blocker, CR = complete response, Cr = creatinine, CsA = cyclosporin, F = female, HBV = hepatitis B virus, HCQ = hydroxychloroquine, M = male, MCTD = mixed connective tissue disease, MMF = mycophenolate mofetil, NR = not reported, PBC = primary biliary cirrhosis, PIG = podocyte infolding glomerulopathy, PR = partial response, PSL = prednisolone, pSS = primary Sjogren syndrome, RA = rheumatoid arthritis, RTX = rituximab, SLE = systemic lupus erythematosus, SS = Sjogren syndrome, UCTD = undifferentiated connective tissue disease.

**Table 2:** Pathological characteristics of PIG cases reported in the literature.

Case No.	Sex	Age	Pathology						
			LM diagnosis	Mesangial deposit	GBM thickening	FPE	Microspheres	Microtubules	IF staining
1^2^	M	31	MGA	Mild	Present	Present	Present	Absent	All negative
2^2^	F	37	LN class II	Mild	Present	Present	Present	Present	G, A, C3, C1q
3^2^	F	40	LN class II	Mild	Present	Present	Present	Present	G, A, C3, C1q
4^2^	F	30	LN class II	Mild	Present	Present	Present	Absent	G, A, C3, C1q, C5b-9
5^2^	F	61	LN class II	Mild	Present	Present	Present	Absent	G, M, C1q
6^2^	F	29	MN	Absent	Present	Present	Present	Absent	All negative
7^2^	F	46	MN	Absent	Present	Present	Present	Absent	All negative
8^2^	F	27	LN class V	Absent	Present	Present	Present	Present	G, A, M, C3, C1q, C5b-9
9^2^	M	53	MN	Absent	Present	Present	Present	Present	All negative
10^2^	F	23	LN class V	Mild	Present	Present	Present	Present	G
11^2^	F	31	LN class V	Absent	Present	Present	Present	Present	G
12^2^	F	24	LN class V	Mild	Present	Present	Present	Present	G, M, C1q
13^2^	M	49	MPGN (type 3)	Present	Present	Present	Absent	Present	G, A
14^2^	F	20	MGA	Absent	Present	NR	Present	Absent	G
15^2^	F	47	MGA	Absent	Present	NR	Present	Absent	G, A, M
16^2^	F	51	MGA	Absent	Present	Present	Present	Absent	All negative
17^2^	F	30	MGA	Absent	Present	Present	Present	Absent	G
18^2^	F	54	FSGS	Present	Present	Present	Present	Absent	All negative
19^2^	F	57	FSGS	Absent	Present	Present	Present	Absent	All negative
20^2^	M	45	FSGS	Present	Present	Present	Present	Present	G,A,C3
21^2^	F	42	FSGS + MN	Absent	Present	Present	Present	Absent	G
22^2^	F	69	MN	Absent	Present	Present	Present	Absent	G,A,M,C3
23^2^	M	46	MN	Absent	Present	Present	Present	Present	G
24^2^	M	59	MN	Absent	Present	Present	Present	Absent	M
25^2^	F	45	MN	Absent	Present	Present	Present	Present	G,A,C3
26^13^	F	14	FSGS	Absent	Absent	Present	Present	Absent	M,C3, C1q
27^14^	M	79	NR	Absent	Present	Present	Present	Absent	All negative
28^15^	F	44	NR	Mild	Present	Present	Present	Present	M
29^16^	F	45	MN	Absent	Present	Present	Present	Absent	G, C3
30^17^	F	27	FSGS	Absent	Present	Present	Present	Absent	M
31^17^	F	23	LN class II	Absent	Present	Present	Present	Absent	M
32	F	34	LN class II	Absent	Present	Present	Present	Present	All negative

A = IgA, F = female, FPE = foot process effacement, FSGS = focal segmental glomerular sclerosis, G = IgG, GBM = glomerular basement membrane, IF = immunofluorescent, LM = light microscopy, LN = lupus nephritis, M = male; M (in IF staining column), IgM, MGA = mild glomerular abnormality, MN = membranous nephropathy, MPGN = membranous proliferative glomerulonephritis, NR = not reported, PIG = podocyte infolding glomerulopathy.

Currently, the pathogenesis of PIG remains unclear. Nakajima et al^[5]^ analyzed extracellular structures, including microspheres and fibrous structures, in various glomerular diseases using immunoelectron microscopy, and found complement or complement fragments, distributed on their membranes. Hinglais et al^[6]^ found that circular granules and striated membrane-like structures expressing the membrane-attacking complex C5b-9 in GBM by immunohistochemistry. This suggests a potential association between PIG and the activation of a specific complement system on podocytes, possibly associated with podocyte injury disrupting matrix biosynthesis and degradation balance, and GBM thickening with increased permeability.^[7,8]^ Feng et al^[3]^ identified mutations in the INF2 gene, known for inherited focal segmental glomerulosclerosis (FSGS).^[9–11]^ Additionally, a case report by Xiong et al^[12]^ showed a novel SMARCAL1 mutation. These suggest that genetic mutations may be involved in the PIG process.

Generally, the clinical outcome for PIG is favorable. The majority of cases (27/32, 84.4%) received immunosuppressive therapy, and achieved at least partial remission. Only a very small number of patients showed disease progression. From this perspective, PIG may be considered a relatively benign disease.

In conclusion, this study reported a case of PIG and SLE. The patient was diagnosed through biopsy, and the disease remained stable after immunosuppressive therapy. The literature review indicates that the relationship between PIG and autoimmune disorders is unclear, and further investigation is needed to understand the specific pathophysiological mechanisms involved. EM remains the gold standard for diagnosing PIG, and it is recommended to thoroughly examine renal biopsies from patients with proteinuria and underlying autoimmune diseases to identify additional cases and comprehend the clinicopathologic features of PIG.

## Acknowledgments

The authors thank Guangzhou University of Chinese Medicine, Dongguan hospital, for its technical assistance for this study.

## Author contributions

**Conceptualization:** Huiqing Zhang.

**Data curation:** Huiqing Zhang, Jie Lin, Hanqi Lu, Yunliang Zhong, Bin Kuang.

**Formal analysis:** Lie Deng, Qiang Li.

**Methodology:** Huiqing Zhang, Jie Lin, Hanqi Lu, Yunliang Zhong, Lie Deng, Bin Kuang.

**Supervision:** Bin Kuang, Qiang Li.

**Writing – original draft:** Huiqing Zhang, Jie Lin.

**Writing – review & editing:** Huiqing Zhang, Jie Lin, Hanqi Lu, Yunliang Zhong, Lie Deng, Bin Kuang, Qiang Li.
